# Evolution of Physicochemical Properties and Volatile Organic Compound Profiles in Pre-Cooked Braised Chicken During Storage

**DOI:** 10.3390/foods15010091

**Published:** 2025-12-29

**Authors:** Hewei Shi, Lichuang Cao, Yaxin Bai, Yu Wang, Sihao Liu, Lishui Chen, Jiansheng Zhao, Shaohua Meng, Junguang Li

**Affiliations:** 1 Food Laboratory of Zhongyuan, Zhengzhou University of Light Industry, Luohe 462300, China; h04116666@163.com (H.S.); wy92@zzuli.edu.cn (Y.W.); lsio642@163.com (S.L.); chenlishui@zyfoodlab.cn (L.C.); 2 College of Food and Bioengineering, Zhengzhou University of Light Industry, Zhengzhou 450001, China; lichuang.cao@zzuli.edu.cn (L.C.); baiyayaxin@163.com (Y.B.); 3 Yunnan Branch, Institute of Medicinal Plant, Peking Union Medical College, Chinese Academy of Medical Sciences, Jinghong 666100, China; 4 Henan Shuanghui Investment and Development Co., Ltd., Luohe 462000, China; zjs4567@163.com (J.Z.); mshua@163.com (S.M.)

**Keywords:** pre-cooked braised chicken, storage time, quality, volatile organic compounds

## Abstract

This study aimed to characterize the physicochemical, structural, and volatile compound changes in commercially sterilized pre-cooked braised chicken (PBC) during storage at 25 °C, using analyses conducted every 30 days from 30 to 180 days. Assessed parameters included microstructure, color, texture, pH, malondialdehyde (MDA) content, Ca^2+^-ATPase activity, and volatile organic compounds (VOCs). Significant quality changes occurred during storage. Specifically, the L* value decreased, and the a* value increased, while hardness, springiness, chewiness, and Ca^2+^-ATPase activity declined. pH increased from 6.01 to 6.59, and MDA content rose from 10.16 to 23.42 nmol/g. 91 VOCs were identified by gas chromatography-ion mobility spectrometry (GC-IMS), comprising 13 alcohols, 18 aldehydes, 18 ketones, 3 acids, 9 esters, 12 hydrocarbons, 6 aromatics, and 12 others. VOC profiles shifted dynamically: key aldehydes and ketones decreased initially, then increased, whereas alcohols, esters, hydrocarbons, and sulfur-containing compounds increased, then decreased. Prolonged storage significantly deteriorated the quality and altered the flavor profile, providing insights for PBC storage.

## 1. Introduction

Global consumption of chicken has risen significantly owing to its high nutritional quality and affordability [1]. Chicken is widely consumed because it typically contains less total fat and cholesterol than common red meats (e.g., pork and beef) and is relatively rich in unsaturated fatty acids, which are considered beneficial for human health [2]. Pre-cooked dishes are fully or partially prepared and packaged products that consumers can heat and eat with minimal additional processing [3,4]. The pre-cooked food industry has experienced remarkable expansion and is expected to continue growing due to consumer demand for convenient, ready-to-eat options. China’s pre-cooked food market is booming, projected to reach a cumulative value of trillion yuan by 2026 with an estimated annual growth rate of 30% [5,6].

Braised chicken, a traditional Chinese dish, is typically made from tender cuts of chicken thighs with mushrooms and exquisite sauces through a standardized process [7]. The pre-cooked braised chicken (PBC) exhibits a golden, glossy appearance, a pronounced aroma, and a tender, juicy mouthfeel. It is rich in high-quality proteins and amino acids that support daily muscle maintenance and metabolic processes, making it a nutritionally balanced and healthful dish. PBC is simply reheated for serving, striking the exceptional convenience-quality balance and aligning perfectly with contemporary consumer demand for both health-conscious and time-efficient meal solutions. The production process for PBC involves tumbling and marinating trimmed chicken thigh meat, followed by sealing the mixture in aluminum foil pouches and subjecting it to high-temperature sterilization. This product has become one of the representative products in the pre-cooked meat dishes.

Quality deterioration of pre-cooked meat dishes during storage is inevitable, primarily due to lipid peroxidation and protein breakdown [8]. It is well known that the volatile flavor profile of meat, including the composition and concentration of each compound, is affected by storage time [9,10]. For instance, Qi et al. [11] demonstrated that the flavor characteristics of stewed chicken significantly improved after freezing. Sun et al. [12] determined the aroma compounds of cooked beef meatballs under different frozen storage durations, and clarified the key aroma components that develop during storage. Prolonged freezing results in the formation of large and irregular extracellular ice crystals, which disrupt protein structures and degrade meat quality [13]. Storage time has a significant effect on meat flavor, and the deterioration of meat quality during storage can be assessed by qualitative and quantitative analyses of volatile compounds [14]. Al-Dalali et al. [15] investigated the effects of long-term frozen storage on marinated raw beef and found that it induces fluctuations in flavor compound concentrations, with lipid degradation emerging as the primary factor influencing the aroma profile. Generally speaking, PBCs are subject to quality changes and flavor dissipation during storage [16].

Although many studies have examined meat quality changes during frozen or chilled storage, the specific effects of the flavor and physicochemical alterations of pre-cooked dishes under ambient storage conditions remain scarce. Consequently, significant knowledge gaps persist regarding the evolution of quality and volatile flavor compounds in reheated pre-cooked dishes, including PBC across different storage durations. To address this gap, this study focuses on pre-packaged meat dishes stored at ambient temperature with different storage periods, aiming to characterize temporal changes and enhance the practical applicability of the research.

This study investigated reheating-induced changes in ambient-stored pre-cooked dishes. PBC was used as a model system to quantify the evolution of physicochemical properties (color, texture, microstructure, pH), biochemical indicators (lipid peroxidation, Ca^2+^-ATPase activity), and VOCs over six months of storage. The study aimed to elucidate quality degradation mechanisms in reheated pre-cooked meats under ambient conditions, thereby establishing a mechanistic basis for optimizing industrial production processes.

## 2. Materials and Methods

### 2.1. Materials

PBC samples were supplied by Henan Shuanghui Investment and Development Co., Ltd. (Luohe, China). The glutaraldehyde reagent was acquired from Beijing Solarbio Science & Technology Co., Ltd. (Beijing, China). The malondialdehyde (MDA) content determination kit and dichloromethane were purchased from Shanghai Macklin Biochemical Technology Co., Ltd. (Shanghai, China). The Ca^2+^-ATPase assay kit was purchased from Jiancheng Bioengineering Institute Co., Ltd. (Nanjing, China). The following n-ketones, comprising 2-butanone, 2-pentanone, 2-hexanone, 2-heptanone, 2-octanone, and 2-nonanone, were purchased from Aladdin Biochemical Technology Co., Ltd. (Shanghai, China).

### 2.2. Sample Preparation

The chickens used in the PBC product produced by Shuanghui were white-feathered broilers sourced from Zhoukou, Henan Province. The processing flow is shown in Supplementary Figure S1. After being sealed in aluminum-foil pouches, the PBC products were sterilized at 115 °C for 55 min to achieve commercial sterility. The product has a shelf life of seven months under ambient storage conditions. PBC images are available in Supplementary Figure S2. Each serving of the PBC product has a net weight of at least 200 g, with chicken meat comprising at least 50% and solids accounting for at least 70% of the product. All samples used in this experiment were produced and supplied by Shuanghui, with approximately 50 packages provided each time. PBC samples were stored in a thermostat (HJU0-X705L00, Haier, Qingdao, China) set to 25 °C to simulate ambient storage conditions. Samples were analyzed at 30-day intervals (30, 60, 90, 120, 150, and 180 days). Prior to analysis, samples were reheated following the manufacturer’s instructions and immersed in a water bath for 8 min. The internal temperature was monitored in real time using a multiplexed temperature checker (AT4508, Anbai, Changzhou, China), and reheating was terminated immediately upon reaching 72.5 °C at the sample center.

#### Experimental Design

To provide a concise overview of the experimental layout, the following summarizes the key conditions and procedures used in this study.

Storage conditions: Commercially sterilized pre-cooked braised chicken (PBC) samples were stored in their original sealed commercial aluminum-foil pouches at 25 °C in a temperature-controlled thermostat.

Storage duration and sampling frequency: Samples were stored for 180 days and sampled at 30-day intervals (30, 60, 90, 120, 150, and 180 days). The first practical sampling point was 30 days post-production because day-0 samples were not available from the commercial supply.

Replicates and sampling unit: At each sampling time point, three independent commercial packages were selected and analyzed. For each analytical method, measurements were performed in triplicate as appropriate; mean ± SD is reported.

Main analyses performed: The study focused on physicochemical and flavor attributes, including microstructure (SEM), color (L*, a*, b*, ΔE, Browning Index), texture profile analysis (TPA: hardness, springiness, chewiness, cohesiveness), pH, lipid oxidation, Ca^2+^-ATPase activity, and volatile organic compounds (VOCs) by GC-IMS. Details for each method are provided in the corresponding subsections.

### 2.3. Color Determination

Color analysis was performed based on a method described by Kusaimah et al. [17]. Reheated chicken thigh samples were blot-dried to remove surface moisture and grease, then trimmed into 8 mm × 8 mm × 8 mm cubes. The L* (lightness), a* (redness), and b* (yellowness) values were measured using a fully automated colorimeter (RM200QC, X-Rite, Grand Rapids, MI, USA). The instrument was calibrated with a standard white calibration plate before each measurement session. Browning Index (BI) can be used to indicate the degree of dullness and browning in color, with its value determined through Formulas (1) and (2). The total color difference ∆E was calculated by Formula (3).

(1) $$X=\frac{a^{*}+1.75L^{*}}{5.645L^{*}+a^{*}-3.012b^{*}}$$

(2) $$BI=\frac{100(X-0.31)}{0.172}$$

(3) $$\Delta E=\sqrt{{\left(\Delta L^{*}\right)}^{2}+{\left(\Delta a^{*}\right)}^{2}+{\left(\Delta b^{*}\right)}^{2}}$$

### 2.4. Texture Profile Analysis (TPA)

TPA was conducted following the method of Huidobro et al. [18] with slight modifications. Reheated samples were equilibrated at room temperature for 3 h, then trimmed into 8 mm × 8 mm × 8 mm cubes. Analysis was performed using a texture analyzer (CTX, Brookfield, MA, USA) equipped with a TA-AACC 36 cylindrical probe in (CTX, Brookfield, MA, USA) TPA mode. Parameters included a test speed of 1 mm/s and 50% deformation. Instrument calibration was performed prior to each testing session.

### 2.5. Scanning Electron Microscope (SEM) of Muscle Fibers

Muscle fiber ultrastructure was examined in accordance with the method of Zhou et al. [19] with modifications. Reheated samples were trimmed into 8 mm × 8 mm × 2 mm tissue blocks and fixed in pre-chilled 2.5% glutaraldehyde at 4 °C for 24 h. Samples underwent sequential ethanol dehydration (10%, 30%, 50%, 70%, 80%, 90%, and absolute ethanol; 10 min per step), followed by freeze-drying (SCIENTZ-12ND, Ningbo, China). Gold sputter-coating was applied prior to imaging. Cross-sectional analysis of muscle fibers was performed using a scanning electron microscope (Nova Nano SEM 450, FEI, Hillsboro, OR, USA) at 350× magnification.

### 2.6. pH Determination

pH measurement was performed, following the method of Wang et al. [20] with slight modifications. Five grams of the reheated sample were homogenized with 45 mL of deionized water at 10,000 rpm for 30 s. The homogenate was equilibrated at room temperature for 30 min, and then centrifuged at 10,000× *g* for 5 min at 4 °C using a benchtop high-speed refrigerated centrifuge (H4-20KR, KeCheng, Changsha, China). pH of the supernatant was measured using a digital pH meter (PHSJ-5, INESA, Shanghai, China).

### 2.7. Determination of MDA Content

MDA content was quantified using a commercial assay kit. Reheated PBC samples (1 g) were homogenized in 10 mL ice-cold extraction buffer and centrifuged at 8000× *g* for 10 min. Subsequently, 0.1 mL aliquots of supernatant were mixed with 0.3 mL of the kit reagent. The reaction mixtures were incubated at 95 °C for 30 min, rapidly cooled in an ice bath, and centrifuged at 10,000× *g* for 10 min at 25 °C. Absorbance of the resulting supernatant was measured at 532 nm and 600 nm. MDA content in lipids was expressed as nanomoles per gram sample. MDA content is calculated according to Formula (4).

(4) $$\mathrm{MDA}=\frac{\frac{\Delta A\;\times \;V_{total}\;\times \;10^{9}}{\varepsilon \;\times \;d}}{\frac{W\;\times \;V_{sample}}{V_{extract}}}$$

where ΔA is the difference in absorbance between the supernatant at 532 nm and 600 nm, V_total_ is the total reaction volume (0.4 mL), ε is the molar absorption coefficient for the MDA (1.55 × 10^5^/L/mol/cm), d is the cuvette pathlength (1 cm), V_extract_ is extraction volume (10 mL), V_sample_ is the added sample volume (0.1 mL), W is the sample mass (1 g), and 10^9^ is the conversion factor, where 1 mol = 10^9^ nmol.

### 2.8. Determination of Ca^2+^-ATPase Activity

One gram of muscle tissue from the PBC samples was homogenized in 9 mL of ice-cold saline (4 °C) at 10,000 rpm for 1 min, maintaining samples on ice. The resulting homogenate was centrifuged at 2500 rpm for 10 min at 4 °C. The supernatant was collected and diluted 10-fold with ice-cold saline. The diluted supernatant was combined with reagents from a commercial Ca^2+^-ATPase assay kit(Jiancheng Bioengineering Institute Co., Ltd., Nanjing, China), following the manufacturer’s instructions. The reaction mixture was incubated in a water bath at 37 °C for 10 min to initiate ATP hydrolysis. Following the phosphorus fixation, the reaction was terminated by the addition of 0.5 mL of terminating agent. The terminated mixture was then incubated at room temperature for 5 min. Subsequently, 200 µL of the supernatant was transferred to a well of a microplate reader-compatible plate. The absorbance was measured spectrophotometrically at a wavelength of 636 nm. The Ca^2+^-ATPase activity is expressed as micromoles of inorganic phosphate released per milligram of protein per hour (µmolPi/mgprot/h). Ca^2+^-ATPase activity is calculated according to Formula (5).

(5) $$ATPase\;activity=\frac{\frac{\Delta A}{A_{standard}}\times C_{standard}\times N\times 6}{C_{pr}}$$

where C_standard_ represents the concentration of the phosphorus standard solution (0.02 µmol/mL), N denotes the dilution factor of the sample in the enzymatic reaction system (2.8), 6 indicates six times the reaction time of 10 min, and C_pr_ represents the protein concentration of the sample.

### 2.9. Analysis of Volatile Compounds by Gas Chromatography-Ion Mobility Spectrometry (GC-IMS)

#### 2.9.1. Extraction of Volatile Flavor Substances via Simultaneous Distillation Extraction (SDE)

For the extraction, 100 g of the PBC sample was placed in a flask and mixed with 300 g of deionized water, then heated to 130 °C. In another flask, 60 mL of dichloromethane was added, and the flask was maintained at 60 °C. Following the initial reflux, the extraction continued for 3 h. The resulting extract was dehydrated by adding an appropriate amount of anhydrous Na_2_SO_4_ at −18 °C for 24 h. The extract was then filtered and concentrated using spin distillation to a volume of 6–8 mL, further filtered through a 0.22 μm membrane (PTFE, INLUCK, Beijing, China), and finally concentrated by nitrogen blowing (EV8, WIGGENS, Straubenhardt, Germany) to 0.5 mL. The final extract was stored at −20 °C until analysis.

#### 2.9.2. Headspace Sampling Conditions

Volatile flavor substances were determined following the methodology of Chen et al. [21]. A 20 µL aliquot of the extract was transferred to a 20 mL headspace vial and underwent thermal equilibration at 80 °C for 15 min under continuous agitation at 500 rpm in an automated sample incubator. The sample was then injected in splitless mode by an autosampler (CTC-PAL 3, CTC Analytics AG, Basel, Switzerland) equipped with a DVB/CAR/PDMS injection needle and a 50/30 µm SPME extraction head, using an injection volume of 200 µL. The temperature of the injection needle was maintained at 85 °C.

#### 2.9.3. Chromatographic Conditions

Chromatographic separation was performed using an MXT™-WAX capillary column (15 m × 0.53 mm × 1.0 µm film thickness; Restek, Bellefonte, PA, USA). High-purity nitrogen (≥99.999%) was employed as the carrier gas. The gas flow rate was programmed as follows: initially maintained at 2.0 mL/min for 2 min, then linearly increased to 10.0 mL/min by 10 min, subsequently to 100.0 mL/min by 20 min, and ultimately to 150.0 mL/min by 40 min. The total chromatographic run time was 40 min, with the injection port maintained at 80 °C.

#### 2.9.4. Ion Mobility Spectrometry (IMS) Conditions

FlavourSpec^®^ gas-phase ion mobility spectrometry (FlavourSpec^®^, G.A.S., Dortmund, Germany) was used to analyze volatile flavor compounds. A tritium (^3^H) source was employed for ionization. The migration tube was 53 mm in length, maintained at 45 °C, and subjected to an electric field strength of 500 V/cm. High-purity nitrogen (purity ≥ 99.999%) was used as the drift gas at a flow rate of 150 mL/min. All measurements were conducted in positive ion mode. A mixed standard containing six ketones was analyzed to construct calibration curves for retention time and retention indices (RIs). The RI of each target compound was then calculated based on these calibration curves.

Identification of target compounds was achieved by comparing their calculated RIs with those in the NIST 2020 GC-RI database and matching their IMS migration time against the IMS database built into VOCal software. The Reporter and Gallery Plot plug-ins in VOCal 0.4.03 were used to generate the three-dimensional plot, two-dimensional differential comparison plot, and fingerprint heatmap of volatile compounds for comparative analysis of VOCs among samples.

### 2.10. Data Analysis

Experiments were triplicated, and the data are presented as mean ± standard deviation. For statistical analysis, one-way ANOVA was applied using SPSS 27.0 software (IBM, Armonk, NY, USA), defining significance at *p* < 0.05. GraphPad Prism 10.0.1 was utilized for graphical analysis and figure preparation (GraphPad, CA, USA).

## 3. Results and Discussion

### 3.1. Physicochemical Properties of PBC During Storage

#### 3.1.1. Variations in Color of PBC

**Figure 1:**
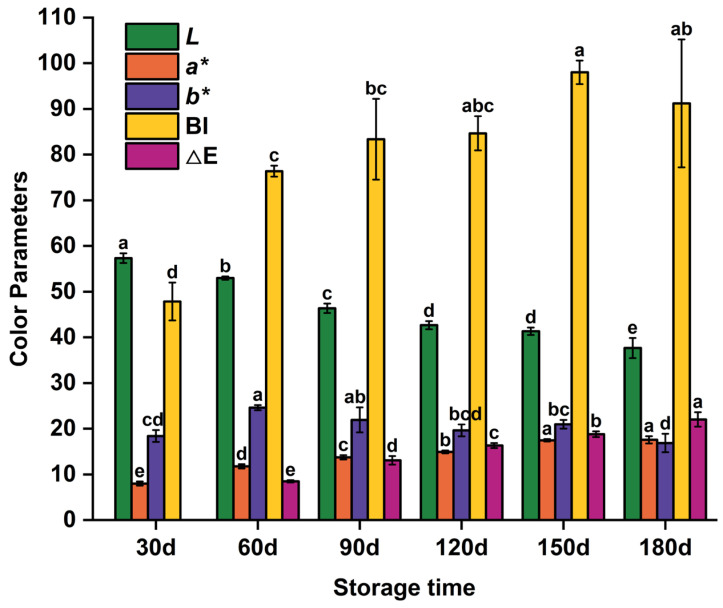
Color parameters evolution of pre-cooked braised chicken during storage. Note: L*, a*, and b* represent the value of color lightness, redness, and yellowness, respectively; BI denotes the browning index; ΔE represents the total color difference. Values with different lowercase letters within the same parameter indicate statistically significant differences at a level of *p* < 0.05.

Color affects consumers’ perception of flavor and is closely related to product quality [21]. In meat products, color changes result from myoglobin denaturation and oxidation, Maillard reactions, and pH variations [22]. Surface color changes in reheated PBC correlated with internal quality alterations during prolonged storage [23]. Figure 1 shows the influence of storage duration on the color of PBC. The L* value, an indicator of lightness, decreased from 57.33 at 30 d to 37.67 at 180 d (*p* < 0.05), indicating progressive darkening of the surface. This darkening likely resulted from oxidation of unsaturated fatty acids, which produced conjugated dienes and carbonyl compounds. Lee et al. [24] observed similar variations. Conjugated dienes may alter the chemical environment around myoglobin or react directly with its heme group, modifying its redox state and affecting meat color. Additionally, carbonyl compounds (e.g., malondialdehyde) can react with amino groups in proteins to form dark polymers (e.g., nigrosine-like compounds), further intensifying surface darkening. Furthermore, intensified protein oxidation during storage reduces muscle fiber water-binding capacity. Prolonged storage combined with reheating exacerbates water loss, increasing light scattering on the sample surface and further decreasing L* values. The a* value, representing redness, increased from 8.00 at 30 d to 17.57 at 180 d (*p* < 0.05), indicating a significant increase in redness. This change may be attributed to myoglobin oxidation. Pujol et al. [25] reported similar findings regarding the effect of myoglobin oxidation on meat color. During storage, myoglobin in chicken meat undergoes oxidation, forming oxymyoglobin and metmyoglobin, which contribute to the redness of the meat color. The b* value, representing yellowness, increased significantly from 30 d to 60 d before showing fluctuations through 180 d, attributable to changes in pigment composition and oxidation intermediates. Wang et al. [26] reported similar results in rabbit meat stored under different temperature conditions. Initially, accumulation of oxidation products increased yellow pigments, whereas subsequent degradation or polymerization of these products at later stages resulted in color fluctuations.

To further quantify browning and overall color change, the BI and the ΔE, with 30 d used as the reference for ΔE, were calculated. BI showed a significant increase from the 30-day baseline and reached its maximum at 150 d; there was no significant difference among BI values at 120 d, 150 d, and 180 d. This suggests that browning progressed markedly during the early-to-mid storage period and then tended to stabilize. ΔE is related to color parameters and can be used to evaluate the effects of different storage times on PBC color. It showed a high value (>5), indicating that color changes are discernible even to inexperienced observers. ΔE increased significantly from 60 d and continued to rise through 180 d (*p* < 0.05), indicating that the overall color difference is likely to be noticeable to consumers compared with the 30 d baseline. These integrated indices (BI and ΔE) are consistent with the single-channel L*, a*, and b* trends and together reinforce the conclusion that ambient storage induces significant color deterioration in reheated PBC.

#### 3.1.2. Textural Properties of PBC During Storage

Texture characterization is essential for both quality control and consumer acceptance. Hardness, springiness, and chewiness are widely used to quantify textural attributes. Hardness refers to resistance to deformation and is governed by myofibrillar protein integrity and water-holding capacity. Springiness represents the sample’s ability to recover its original shape elastically following deformation removal, while chewiness quantifies the energy required to masticate it to a state suitable for swallowing [27]. As shown in Table 1, the hardness, springiness, and chewiness of PBC declined significantly during storage (*p* < 0.05). This decline may result from the denaturation of myofibrillar protein during storage, reducing the structural integrity of muscle fibers. This decline is consistent with Karlović et al. [28], who reported texture softening in prolonged-stored meat products and may be explained by several, potentially co-occurring mechanisms. In contrast, the cohesiveness of PBC remained unaffected by storage duration. First, myofibrillar protein denaturation and oxidative modification weaken the cohesive strength of the myofibrillar network, leading to muscle tissue softening and reduced hardness [29]. Oxidation of myosin can disrupt cross-bridge interactions and weaken structural integrity, resulting in diminished hardness and chewiness. Second, continuous water loss during storage alters water-holding capacity, affecting meat elasticity, which is consistent with the results of Hou et al. [30]. Furthermore, chewiness progressively declines with extended storage duration. This trend may also stem from ongoing lipid oxidation, as lipid oxidation reduces meat tenderness, which is consistent with the results of Zhang et al. [31].

#### 3.1.3. Variations in Microstructure in PBC

**Figure 2:**
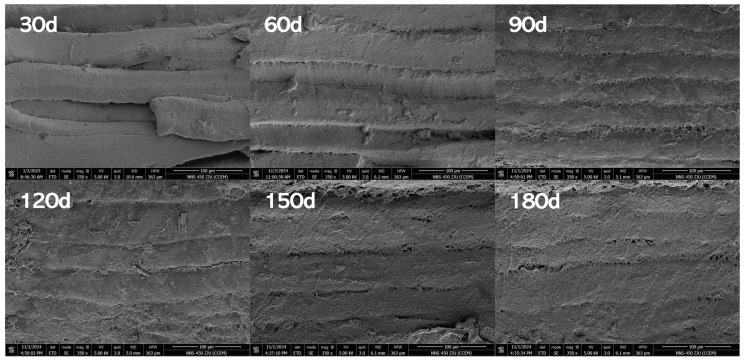
Effects of storage time on the microstructure of pre-cooked braised chicken (under a magnification of 350×).

SEM provides direct visualization of microstructural alterations in reheated PBC during storage. A smaller diameter and tighter organization of myofibrils indicate superior quality [32]. Figure 2 visualizes microstructural transformations in PBC muscle fibers across increasing storage intervals. During the 30–60-day period, the microstructure maintained relative regularity with distinct boundaries, though subtle reductions in overall compactness and hierarchical organization became apparent. From 60 to 90 days, pronounced structural alterations emerged, characterized by significant architectural loosening, boundary blurring, progressive deterioration of hierarchical organization, and compromised integrity. Progressing to 90–120 days, structural deterioration intensified: global architecture became increasingly disordered, regional boundaries indistinct, and muscle fibers exhibited fragmented adhesion. At 120–150 days, fragmentation peaked with near-complete loss of coherent architecture, resulting in fiber dispersion and substantially diminished compactness. By 180 days, microstructural integrity was virtually lost, with fibers displaying severe deterioration characterized by coarse, ill-defined morphology and consequential loss of tissue stability.

This structural progression is primarily driven by lipid oxidation, which generates aldehydes and ketones capable of modifying myofibrillar proteins. Such interactions promote protein denaturation, disrupt myofibrillar organization, and ultimately compromise microstructural integrity, leading to quality deterioration [33]. Concurrently, protein oxidation may induce cross-linking or fragmentation of protein molecules, further altering tertiary structures and compromising microstructural integrity [34]. These mechanisms align with documented patterns of myofibrillar deterioration during storage, including progressive loosening of architecture and expansion of inter-fiber gaps [35]. Consistent with this, Lu et al. [36] reported temperature and duration-dependent increases in myofibrillar spacing in bovine muscle. While these studies attribute structural changes to proteolytic degradation during storage, the observed blurring of muscle fiber boundaries in PBC specifically stems from intensified oxidative processes triggered by reheating. Notably, this phenomenon is compounded by the lateral expansion of muscle fibers into adjacent spaces, a distinct mechanistic contribution identified in this study.

#### 3.1.4. Analysis of pH, MDA, and Ca^2+^-ATPase Activity

**Figure 3:**
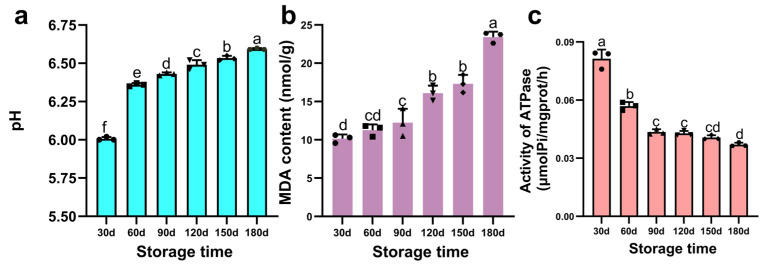
Changes in pH, malondialdehyde (MDA) content, and Ca^2+^-ATPase activity of pre-cooked braised chicken during storage. (**a**) pH value, (**b**) MDA content, (**c**) Ca^2+^-ATPase activity. Note: Different lowercase letters above bars indicate statistically significant differences (*p* < 0.05).

The storage stability of PBC was evaluated by monitoring pH changes. As shown in Figure 3a, pH increased significantly with storage duration, likely ascribed to protein degradation and subsequent generation of amines, ammonia, and other alkaline nitrogenous compounds [37]. These findings align with Jung et al. [38], who documented a significant pH increase in chicken breast meat across various broiler breeds during 1–7 days of storage. This phenomenon is primarily attributed to endogenous enzymatic proteolysis, which liberates amines and ammonia, elevating pH through their accumulation [39]. Similarly, Xiao et al. [40] reported pH values exceeding 6.0 in hindquarter muscles of Hengshan goats following extended storage under different temperatures. Furthermore, lipid peroxidation-derived aldehydes and ketones may function as contributory factors, potentially influencing acid-base equilibria and indirectly modulating pH.

Lipids in meat products undergo progressive oxidative deterioration during storage, generating reactive secondary products that progressively degrade organoleptic properties. The MDA content serves as a well-established quantitative indicator of lipid peroxidation severity, where elevated concentrations correlate directly with the advancement of oxidative damage. As illustrated in Figure 3b, lipids in PBC exhibited progressive peroxidation throughout storage, leading to the accumulation of MDA as the principal secondary product. The MDA content gradually increased to 12.24 nmol/g during the initial 90-d period, followed by a significant rise to 16.10 nmol/g at day 120, and ultimately reaching 23.42 nmol/g by day 180. This accelerating trend aligns with studies documenting lipid oxidation kinetics in processed meats. Kim et al. [41] similarly observed progressive oxidation in marinated, steamed pork tenderloin over 8 weeks of storage. Furthermore, Roldan et al. [42] established that lipid oxidation dynamics are critically influenced by both storage duration and processing conditions.

Myofibrillar proteins are critical components of meat products and can affect the integrity of meat ultrastructure. As a key indicator of myofibrillar stability during storage, Ca^2+^-ATPase activity reflects the structural integrity of myosin within myofibrillar protein. As shown in Figure 3c, Ca^2+^-ATPase activity decreased continuously (*p* < 0.05) throughout storage in PBC. This decline is linked to oxidative denaturation of myofibrillar proteins, consistent with observations by Zhang et al. [43] in both cured and uncured pork during storage. Furthermore, free radicals generated during concomitant protein and lipid oxidation likely mediate oxidative modifications of Ca^2+^-ATPase, inducing conformational changes that impair its catalytic function. Critically, the observed Ca^2+^-ATPase activity trajectory exhibits strong concordance with trends in pH evolution, ultrastructural alterations, and textural degradation, collectively indicating systemic deterioration driven by oxidative processes.

### 3.2. Volatile Profiles of PBC During Storage

#### 3.2.1. Identification and Characterization of Volatile Compounds in PBC

GC-IMS was applied to characterize volatile flavor compounds and track their variation patterns in PBC during storage. The complete VOC dataset is available in Supplementary Table S1. A total of 91 VOCs was identified, categorized as 13 alcohols, 18 aldehydes, 18 ketones, 3 acids, 9 esters, 12 hydrocarbons, 6 aromatics, and 12 miscellaneous compounds. Notably, aldehydes, primarily generated during unsaturated fatty acid degradation, exhibit low sensory thresholds and are recognized as major contributors to chicken flavor [44]. However, the overall flavor profile of PBC arises from synergistic interactions among volatile and non-volatile compounds [45].

**Figure 4:**
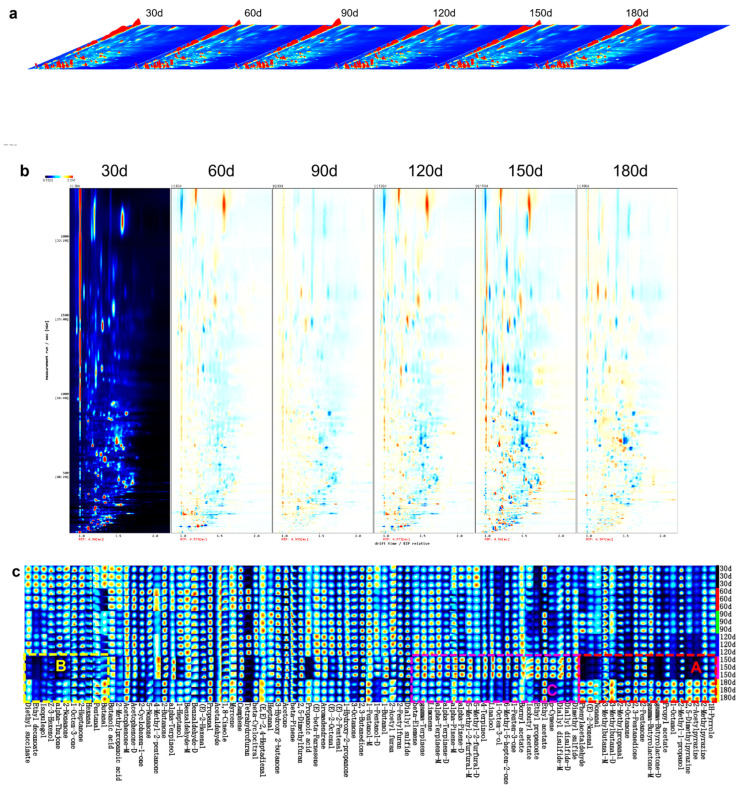
GC-IMS analysis of volatile compounds in pre-cooked braised chicken during storage. (**a**) Three-dimensional plot, (**b**) Two-dimensional differential comparison plot, and (**c**) Fingerprint heatmap of volatile organic compounds.

Three-dimensional visualization of VOCs in PBC across storage intervals is presented in Figure 4a. This plot spatially resolves VOC profiles through orthogonal dimensions: migration time (*X*-axis), retention time (*Y*-axis), and signal intensity (*Z*-axis), where peak magnitude correlates directly with compound abundance. The figure clearly visualizes the differences in both the composition and concentration of VOCs during storage.

For comparative analysis, a two-dimensional differential plot was generated using the 30-day sample as the reference. In this framework, the blue background denotes the reference profile; the red vertical line at 1.0 marks the normalized reactive ion peak (RIP); points distributed along RIP represent detected VOCs [46]. The horizontal and vertical axes correspond to normalized drift time and GC retention time, respectively, while color intensity reflects relative concentration. The differential comparison plot (Figure 4b) was constructed by subtracting the reference profile from profiles at different storage times. Resulting white areas indicate VOC parity between target and reference samples, red areas denote elevated concentrations in target samples, and blue areas reflect reduced concentrations [47]. Figure 4b reveals fluctuating VOCs concentrations spanning relative migration times of 1.0–2.0 ms and retention times of 0–2500 s. Specific VOCs increased with extended storage, while others diminished or stabilized. These variations are attributed to storage-induced biochemical processes such as lipid peroxidation, protein oxidation, denaturation, hydrolysis, and Maillard reactions, which collectively modified the VOC profiles of PBC [48].

#### 3.2.2. Comparative Analysis of VOCs Fingerprints in PBC During Storage

Although three-dimensional and two-dimensional differential comparison plots revealed VOC differences across storage periods, they lacked compound-level resolution. In contrast, the fingerprint heatmap provided comprehensive compound-specific data and effectively highlighted storage-dependent variations [49]. Figure 4c illustrates this VOCs fingerprint heatmap for PBC samples: rows represent all informative peaks per sample, while columns correspond to individual volatile compounds across storage intervals. The heatmap was segmented into three regions based on concentration dynamics.

In region A, concentrations of phenylacetaldehyde, (E)-2-nonenal, nonanal, 2-methylpropanal, and 3-methylbutanal decreased during the initial 150 days. This reduction may be attributed to aldehyde instability, as these compounds are prone to oxidation, forming carboxylic acids. For example, 2-methylpropanal oxidizes to 2-methylpropanoic acid, which may subsequently undergo esterification with endogenous alcohols to form esters [50]. Although non-enzymatic browning pathways like Maillard reactions are thermally favored, they proceed slowly under ambient storage. Early Maillard stages involve aldehyde reactions with protein-derived ammonia-containing compounds, forming Schiff base intermediates that may alter system pH. Advanced Maillard stages promote aldehyde transformations through aldol condensation (e.g., nonanal to (E)-2-nonenal) or polymerization into melanoidin pigments that significantly influence PBC coloration. Elevated temperatures also enhance aldehyde volatilization, suggesting volatile losses during prolonged ambient storage and reheating contribute to depletion. Given aldehydes’ critical role in imparting characteristic chicken aroma, their decline attenuates or modifies the meat flavor. Notably, aldehyde concentrations increased again at 180 days. The increase is attributable to two primary mechanisms: (1) ester cleavage yielding short-chain aldehydes, (2) extensive lipid peroxidation generating additional aldehydes [51]. Furthermore, extended storage facilitates gradual Maillard reactions between amino acids and reducing sugars, enabling aldehyde accumulation, while Strecker degradation of amino acids directly generates aldehydes [52]. For instance, proteolysis-derived phenylalanine undergoes decarboxylation and deamination to form phenylacetaldehyde.

In region B, concentrations of 2-nonanone, α-thujone, Z-3-hexenol, isopulegol, ethyl decanoate, and diethyl succinate in PBC gradually decreased during the initial 150 days of storage. Notably, 2-nonanone and α-thujone contribute thujonic and fresh sensory attributes [53]. The reduction of 2-nonanone and α-thujone likely reflects either volatility-driven losses during ambient storage or oxidative cleavage yielding smaller ketones. Additionally, their carbonyl groups may react with amino-containing compounds to form heterocyclic compounds or undergo condensation with other carbonyls (e.g., aldehydes); prolonged storage may further facilitate α-thujone’s structural rearrangement to α-pinene. Conversely, the levels of 2-nonanone and α-thujone increased at 180 days, potentially through two pathways: (i) secondary lipid oxidation generating novel ketones, or (ii) reversion reactions of oxidation products. Ketogenesis via amino acid degradation and Maillard pathways may also contribute to aroma modulation [54]. The reduction in Z-3-hexenol and isopulegol may result from esterification with carboxylic acids derived from aldehyde oxidation [55]. Similarly, ethyl decanoate and diethyl succinate underwent hydrolysis or oxidative degradation during early storage but increased at 180 days, presumably due to esterification processes during extended storage [56].

In region C, concentrations of sulfur-containing compounds, linalool, 4-terpineol, hydrocarbons, and p-cymene increased gradually during the initial 150 days of storage. This trend likely stems from commercial sterilization-induced disruption of spice-derived plant cell walls in PBC, facilitating gradual compound release from cellular matrices during extended storage [57]. Elevated alcohol levels may originate from gluconeogenesis, lipid oxidation, and amino acid decarboxylation/dehydrogenation [54], while hydrocarbon accumulation derives from fatty acid cleavage and degradation [58]. The initial increase in sulfur-containing compounds primarily arises from sulfur amino acid degradation [59]. Conversely, their decline at 180 days reflects either radical-mediated oxidative cleavage of sulfides or interactions between lipid peroxidation products and sulfides, yielding novel compounds. Linalool and 4-terpinenol may degrade via oxidation to ketones or through esterification with amino acids, forming esters. Hydrocarbon reductions are attributed to oxidative fragmentation and volatilization, while the decrease in p-cymene results from volatilization combined with oxidation of its unsaturated structure to peroxides or isomerization to alternative terpenoids. 1-Octen-3-ol accumulated progressively during the initial 150 days via fatty acid oxidation, contributing mushroom aromas that modulate overall flavor [60,61]. Its subsequent decrease may reflect further oxidation to ketones (e.g., 1-octen-3-one) or volatilization. Ethyl propanoate, isobutyl acetate, and bornyl acetate increased during the initial 150 days through acid-alcohol esterification, but decreased by 180 days due to either (i) hydrolytic cleavage followed by aldehyde-forming oxidation or (ii) radical-induced degradation during lipid oxidation.

## 4. Conclusions

This study comprehensively evaluated physicochemical and flavor profile alterations in PBC during storage. Progressive deterioration was observed in color parameters, with a significant decrease in L* value and an increase in a* value (*p* < 0.05). Texture profile analysis demonstrated a remarkable decline in hardness, springiness, and chewiness, concomitant with microstructural degradation as observed by SEM: myofibrils exhibited surface roughening, loss of structural integrity, and increasingly indistinct interfacial boundaries. Biochemical assays revealed a continuous rise in pH and MDA content (*p* < 0.05), concomitant with a significant reduction in Ca^2+^-ATPase activity (*p* < 0.05), collectively indicating declining myofibrillar protein stability. Volatile compound analysis by GC-IMS identified 91 flavor substances, with three- and two-dimensional profiles confirming dynamic changes throughout storage. Aldehydes and ketones exhibited an initial decline prior to an increase during prolonged storage, whereas alcohols, esters, hydrocarbons, and sulfur-containing compounds showed an initial increase followed by a subsequent reduction. These observed changes reflect the dynamic evolution of flavor profiles in stored PBC. However, the complex reaction pathways governing VOCs transformations in pre-cooked meat products remain incompletely understood, necessitating further mechanistic studies to elucidate the underlying biochemical and oxidative processes.

## Figures and Tables

**Table 1:** Effect of storage time on textural properties.

Storage Time (d)	Hardness (g)	Springiness (mm)	Chewiness (g·mm)	Cohesiveness
30	2154.00 ± 218.28 ^a^	3.23 ± 0.25 ^a^	2775.33 ± 871.61 ^a^	0.39 ± 0.05 ^a^
60	1439.00 ± 29.72 ^b^	2.49 ± 0.30 ^b^	1449.33 ± 260.08 ^b^	0.41 ± 0.05 ^a^
90	1146.67 ± 39.31 ^c^	2.24 ± 0.15 ^b^	838.33 ± 66.03 ^bc^	0.33 ± 0.03 ^a^
120	1026.67 ± 28.50 ^cd^	1.73 ± 0.06 ^c^	1041.67 ± 80.36 ^bc^	0.59 ± 0.07 ^a^
150	871.00 ± 74.05 ^d^	1.53 ± 0.06 ^c^	527.67 ± 181.75 ^c^	0.40 ± 0.15 ^a^
180	551.33 ± 141.37 ^e^	1.46 ± 0.06 ^c^	310.00 ± 95.54 ^c^	0.40 ± 0.16 ^a^

Note: Means within the same column with different letters differ significantly (*p* < 0.05).

## Data Availability

The original contributions presented in the study are included in the article and Supplementary Material; further inquiries can be directed to the corresponding author.

## Supplementary Materials

The following supporting information can be downloaded at https://www.mdpi.com/article/10.3390/foods15010091/s1. Figure S1: PBC production flowchart; Figure S2: PBC product image; Table S1: Detailed information on volatile compounds in PBC characterized by GC-IMS.

## References

[1] Hwang C.C., Huang Y.R., Hsieh C.D., Lee Y.C. (2024). Developing novel microwave-assisted induction heating (MAIH) technology for heating in-packaged ready-to-eat chicken breast products. Innov. Food Sci. Emerg. Technol..

[2] Yang C.F., Lv D.L., Wang G.Y., Liu J., Du Y.F., Tang S., Tan J.Y., Liao G.Z. (2025). Comprehensive analysis of changes in taste substances during the processing of Wuding chicken based on HPLC, LC-ESI-MS/MS, and electronic tongue. Int. J. Gastron. Food Sci..

[3] Xu Y., Hu Y., Lan H., Zhang J.H., Gao Y.P., Deng S.G. (2025). Comparative analysis of quality and flavor profiles in raw and pre-cooked large yellow croaker (*Larimichthys crocea*) meat post freezing and reheating. Food Chem..

[4] Huang M.Q., Adhikari B., Lv W.Q., Xu J.C. (2024). Application of novel-assisted radio frequency technology to improve ready-to-eat foods quality: A critical review. Food Biosci..

[5] Fang M., Ke J.Y., Wang Z.J., Fu Q., Yang Q., Xu L., Lu Y.P., Jiang X.M., Wu Y.N., Liu X. (2025). Preparation of sulfonic acid functionalized metal organic frameworks and their application in the online solid phase extraction of parabens and sulfonamides in pre-cooked foods. Food Chem. X.

[6] Ping C.Y., Hu H.L., Bi J.C., Li X., Yi Y.W., Qiao M.F. (2025). Identification of volatile indicators for quality deterioration of Yu-Shiang Shredded pork after reheating based on HS-GC-IMS and intelligent sensory evaluation. Int. J. Gastron. Food Sci..

[7] Lei Y., Zhang Y.L., Cheng Y.Q., Huang J.C., Huang M. (2023). Monitoring and identification of spoilage-related microorganisms in braised chicken with modified atmosphere packaging during refrigerated storage. Food Sci. Hum. Wellness.

[8] Tikk K., Haugen J.E., Andersen H.J., Aaslyng M.D. (2008). Monitoring of warmed-over flavour in pork using the electronic nose—Correlation to sensory attributes and secondary lipid oxidation products. Meat Sci..

[9] Liu Z.J., Huang Y.Q., Kong S.S., Miao J.J., Lai K.Q. (2023). Selection and quantification of volatile indicators for quality deterioration of reheated pork based on simultaneously extracting volatiles and reheating precooked pork. Food Chem..

[10] Xie Q.S., Xu B.C., Xu Y., Yao Z., Zhu B.W., Li X.F., Sun Y. (2022). Effects of different thermal treatment temperatures on volatile flavour compounds of water-boiled salted duck after packaging. LWT-Food Sci. Technol..

[11] Qi J., Xu Y., Zhang W.W., Xie X.F., Xiong G.Y., Xu X.L. (2021). Short-term frozen storage of raw chicken meat improves its flavor traits upon stewing. LWT-Food Sci. Technol..

[12] Sun Y.W., Zhang Y., Song H.L. (2021). Variation of aroma components during frozen storage of cooked beef balls by SPME and SAFE coupled with GC-O-MS. J. Food Process. Pres..

[13] Li J.G., Sun C.H., Yue X.N., Ma W.C., Wang Y., Zhao J.S., Zhu G.S., Bai Y.H. (2025). Ultrasound-assisted immersion freezing improves the digestion properties of beef myofibrillar protein. Food Chem. X.

[14] Xu L.N., Xu X.L., Huang M.Y., Xu Y.J. (2025). UV responded modified polyvinyl alcohol bio-active films with oregano essential oil microcapsules: Microbial control and sensory quality preservation for ready-to-eat chicken breast. Food Control.

[15] Al-Dalali S., Li C., Xu B.C. (2022). Effect of frozen storage on the lipid oxidation, protein oxidation, and flavor profile of marinated raw beef meat. Food Chem..

[16] Lei Y., Huang J.C., Cheng Y.Q., Zhang Y.L., Huang T.R., Huang M. (2022). Changes in bacterial communities and the volatilome of braised chicken with different packaging stored at 4 °C. Food Res. Int..

[17] Kusaimah M., Oladipupo Q.A., Ume R., Khaja M., Hassan M.H., Nilesh P.N., Sajid M. (2023). A Comparative Study on Changes in Protein, Lipid and Meat-Quality Attributes of Camel Meat, Beef and Sheep Meat (Mutton) during Refrigerated Storage. Animals.

[18] Huidobro D.R.F., Miguel E., Blázquez B., Onega E. (2005). A comparison between two methods (Warner–Bratzler and texture profile analysis) for testing either raw meat or cooked meat. Meat Sci..

[19] Zhou Y.J., Hu M.Q., Wang L. (2022). Effects of different curing methods on edible quality and myofibrillar protein characteristics of pork. Food Chem..

[20] Wang T., Jin Y.M., Zhang X., Yang N., Xu X.M. (2024). Effect of Static Magnetic Field on the Quality of Pork during Super-Chilling Storage. Foods.

[21] Yan K.L., Kong J.X., Yu L.M., Yang J., Zeng X.F., Bai W.D., Qian M., Dong H. (2025). Flavor evolution and identification of the warmed-over flavor (WOF) in pre-cooked goose meat by means of HS-SPME-GC-MS and GC-IMS. Food Chem..

[22] José S.D.P., Gázquez A., Jorge R.C. (2012). Physico-chemical, textural and structural characteristics of sous-vide cooked pork cheeks as affected by vacuum, cooking temperature, and cooking time. Meat Sci..

[23] Li Y., Liang S., Ye G.D., Zhang M., Feng S.S., Wang Z.K., Zhang Q.Y., Sun C.X. (2023). Effects of different sterilization methods on sensory quality and lipid oxidation of Dezhou braised chicken. Food Sci. Technol..

[24] Lee G.Y., Lim K.J., Lee Y.H., Shin H.S. (2024). Development of a Freshness Indicator for Assessing the Quality of Packaged Pork Products during Refrigerated Storage. Foods.

[25] Pujol A., Ospina-E J.C., Alvarez H., Muñoz D.A. (2023). Myoglobin content and oxidative status to understand meat products’ color: Phenomenological based model. J. Food Eng..

[26] Wang Z.M., Tu J.C., Zhou H., Lu A., Xu B.C. (2021). A comprehensive insight into the effects of microbial spoilage, myoglobin autoxidation, lipid oxidation, and protein oxidation on the discoloration of rabbit meat during retail display. Meat Sci..

[27] Chen J., Yang X.Y., Xia X.L., Wang L., Wu S.Y., Pang J. (2022). Low temperature and freezing pretreatment for konjac glucomannan powder to improve gel strength. Int. J. Biol. Macromol..

[28] Karlović S., Ježek D., Blažić M., Tripalo B., Brnčić M., Bosiljkov T., Šimunek M. (2009). Influence of refrigeration and ageing time on textural characteristics of fresh meat. Croat. J. Food Sci. Technol..

[29] Yan J., He S.C., Chen L.L., Chen H., Ouyang K.H., Wang W.J. (2024). Effect of gelatin-chitosan-*Cyclocarya paliurus* flavonoids edible coating film on the preservation of chilled beef. LWT-Food Sci. Technol..

[30] Hou F.F., Zhao H.L., Yan L.T., Li S., Chen X.N., Fan J.F. (2023). Effect of CO_2_ on the preservation effectiveness of chilled fresh boneless beef knuckle in modified atmosphere packaging and microbial diversity analysis. LWT-Food Sci. Technol..

[31] Zhang B., Liu Y., Wang H.H., Liu W.H., Cheong K.L., Teng B. (2021). Effect of sodium alginate-agar coating containing ginger essential oil on the shelf life and quality of beef. Food Control.

[32] Sotelo I., Pérez-Munuera I., Quiles A., Hernando I., Larrea V., Lluch M.A. (2004). Microstructural changes in rabbit meat wrapped with *Pteridium aquilinum* fern during postmortem storage. Meat Sci..

[33] Du J.J., Cao J.X., Zhou C.Y., Pan D.D., Geng F., Wang Y. (2022). Insight into the mechanism of myosin-fibrin gelation induced by non-disulfide covalent cross-linking. Food Res. Int..

[34] Kong D.W., Han R.W., Yuan M.D., Xi Q., Du Q.J., Li P., Yang Y.X., Applegate B., Wang J. (2023). Ultrasound combined with slightly acidic electrolyzed water thawing of mutton: Effects on physicochemical properties, oxidation and structure of myofibrillar protein. Ultrason. Sonochemistry.

[35] Hughes J., Clarke F., Purslow P., Warner R. (2017). High pH in beef longissimus thoracis reduces muscle fibre transverse shrinkage and light scattering which contributes to the dark colour. Food Res. Int..

[36] Lu X., Zhang Y.M., Xu B.C., Zhu L.X., Luo X. (2020). Protein degradation and structure changes of beef muscle during superchilled storage. Meat Sci..

[37] Zhang H.Y., Wu J.J., Guo X.Y. (2016). Effects of antimicrobial and antioxidant activities of spice extracts on raw chicken meat quality. Food Sci. Hum. Wellness.

[38] Jung Y., Oh S., Lee S., Lee H.J., Choo H.J., Jo C., Nam C., Lee J.H., Jang A. (2025). Characterization of meat quality, storage stability, flavor-related compounds, and their relationship in Korean Woorimatdag No. 2 chicken breast meat during cold storage. Poultry Sci..

[39] Lee D., Lee H.J., Jung D.Y., Kim H.J., Jang A., Jo C. (2022). Effect of an animal-friendly raising environment on the quality, storage stability, and metabolomic profiles of chicken thigh meat. Food Res. Int..

[40] Xiao Y., Zhao J., Zhang X.R., Jiao Y., Liu Y.F. (2023). Analysis of quality changes of Hengshan goat hindquarter meat at four storage temperatures. J. Food Compos. Anal..

[41] Kim Y.A., Ba H.V., Hwang I. (2019). Effects of traditional sauce type and storage time on quality characteristics, shelf-life and flavor compounds of marinated pork cooked by sous vide method. Food Sci. Anim. Resour..

[42] Roldan M., Antequera T., Armenteros M., Ruiz J. (2014). Effect of different temperature–time combinations on lipid and protein oxidation of sous-vide cooked lamb loins. Food Chem..

[43] Zhang Y., Li H., Zhang Y.J., Wang L.G., Zhang P.C., Jia J.L., Peng H.C., Qian Q., Zhang J.M., Pan Z.L. (2022). Storage stability and flavor change of marinated pork. Foods.

[44] Yin X.Y., Lv Y.C., Wen R.X., Wang Y., Chen Q., Kong B.H. (2021). Characterization of selected Harbin red sausages on the basis of their flavour profiles using HS-SPME-GC/MS combined with electronic nose and electronic tongue. Meat Sci..

[45] Guan H.N., Zhang W.X., Tian Y.L., Leng S.Q., Zhao S.F., Liu D.Y., Diao X.Q. (2025). Analysis of the flavor profile of chicken white soup with varying fat addition using GC–MS, GC-IMS and E-nose combined with E-tongue. Food Chem. X.

[46] Sun P.Z., Lin S.Y., Li X.R., Li D.M. (2024). Different stages of flavor variations among canned Antarctic krill (*Euphausia superba*): Based on GC-IMS and PLS-DA. Food Chem..

[47] Guan H.N., Yang C., Tian Y.L., Feng C.M., Gai S.M., Liu D.Y., Diao X.Q. (2023). Changes in stability and volatile flavor compounds of self-emulsifying chicken soup formed during the stewing process. LWT-Food Sci. Technol..

[48] Han D., Zhang C.H., Fauconnier M.L., Jia W., Wang J.F., Hu F.F., Xie D.W. (2021). Characterization and comparison of flavor compounds in stewed pork with different processing methods. LWT-Food Sci. Technol..

[49] Bassey A.P., Boateng E.F., Zhu Z.S., Zhou T.M., Nasiru M.M., Guo Y.P., Dou H., Ye K.P., Li C.B., Zhou G.H. (2022). Volatilome evaluation of modified atmosphere packaged chilled and super-chilled pork loins using electronic nose and HS-GC-IMS integration. Food Packag. Shelf Life.

[50] Xu Z.L., Chen J.L., Shi X.W., Wang B., Zheng X.C., Zheng X.J. (2020). Characteristic physicochemical indexes and flavor compounds in Xinjiang Kazak cheese during ripening. Food Biosci..

[51] Shahidi F., Rubin L.J., D’Souza L.A., Teranishi R., Buttery R.G. (2009). Meat flavor volatiles: A review of the composition, techniques of analysis, and sensory evaluation. Crit. Rev. Food Sci. Nutr..

[52] García C., Berdagué J.J., Antequera T., López-Bote C., Córdoba J.J., Ventanas J. (1991). Volatile components of dry cured Iberian ham. Food Chem..

[53] Huan Y.J., Zhou G.H., Zhao G.M., Xu X.L., Peng Z.Q. (2005). Changes in flavor compounds of dry-cured Chinese Jinhua ham during processing. Meat Sci..

[54] Zhang L., Hu Y.Y., Wang Y., Kong B.H., Chen Q. (2021). Evaluation of the flavour properties of cooked chicken drumsticks as affected by sugar smoking times using an electronic nose, electronic tongue, and HS-SPME/GC-MS. LWT-Food Sci. Technol..

[55] Hwang H.S., Ball J.C., Doll K.M., Anderson J.E., Vermillion K. (2020). Investigation of polymers and alcohols produced in oxidized soybean oil at frying temperatures. Food Chem..

[56] Carrapiso A.I., Noseda B., García C., Reina R., Pulgar J.S.D., Devlieghere F. (2015). SIFT-MS analysis of Iberian hams from pigs reared under different conditions. Meat Sci..

[57] Huang L.H., Ho C.T., Wang Y. (2021). Biosynthetic pathways and metabolic engineering of spice flavors. Crit. Rev. Food Sci. Nutr..

[58] Meng Q., Zhou J.W., Gao D., Xu E.B., Guo M.M., Liu D.H. (2022). Desorption of nutrients and flavor compounds formation during the cooking of bone soup. Food Control.

[59] Pérez S.C., Carballo J., Fulladosa E., Garcia-Perez J.V., Benedito J., Lorenzo J. (2018). Effect of proteolysis index level on instrumental adhesiveness, free amino acids content and volatile compounds profile of dry-cured ham. Food Res. Int..

[60] Chen Y., Li P., Liao L.Y., Qin Y.Y., Jiang L.W., Liu Y. (2021). Characteristic fingerprints and volatile flavor compound variations in Liuyang Douchi during fermentation via HS-GC-IMS and HS-SPME-GC-MS. Food Chem..

[61] Zhan F.L., Sun L.X., Zhao G.M., Li M.Y., Zhu C.Z. (2022). Multiple technologies combined to analyze the changes of odor and taste in Daokou braised chicken during processing. Foods.

