# Supplementary material for: Evolution of Physicochemical Properties and Volatile Organic Compound Profiles in Pre-Cooked Braised Chicken During Storage
Source: Foods. 2025 Dec 29;15(1):91. doi: 10.3390/foods15010091 (PMC12785967; doi:10.3390/foods15010091)
Supplement: Supplementary file 1 [file foods-15-00091-s001.zip › Table S1.pdf]

Supplementary Table S1. Detailed information of volatile compounds in PBC characterized by GC-IMS.

No.	Compounds	CAS	Retention index	Retention Time (s)	Drift Time (a.u.)	Odor Descriptors
Alcohols						
1	α-Terpineol	98-55-5	1673.1	1500.316	1.21655	pine terpenoid, citrus, woody
2	Isopulegol	89-79-2	1661.1	1455.974	1.40116	camphor, mint, rose leaves
3	4-Terpineol	562-74-3	1561.8	1135.466	1.21937	pepper, woody, moldy soil
4	Linalool	78-70-6	1533.4	1057.344	1.21937	citrus, rose, woody
5	1-Octanol	111-87-5	1541.1	1077.997	1.47585	citrus, sweet, herbs
6	1-Heptanol	111-70-6	1442.7	842.537	1.3945	grape, fruity, wine
7	Z-3-Hexenol	928-96-1	1361.2	687.044	1.50569	green, herb
8	1-Pentanol-M	71-41-0	1257.6	517.714	1.25269	balsamic
9	1-Pentanol-D	71-41-0	1257.6	517.714	1.51511	balsamic
10	1,8-Cineole	470-82-6	1200.6	432.559	1.72266	camphor, refreshing herbal
11	1-Butanol	71-36-3	1137.5	348.022	1.39057	wine
12	2-Methyl-1-propanol	78-83-1	1103.3	307.978	1.34865	fresh, alcoholic, leather
13	1-Octen-3-ol	3391-86-4	1448.2	854.169	1.16921	mushroom, lavender, rose
Aldehydes						
1	β-Cyclocitral	432-25-7	1653.0	1426.513	1.31382	fruity, green, mint
2	5-Methyl-2-furfural-M	620-02-0	1602.1	1256.057	1.13221	spices, caramel wood
3	5-Methyl-2-furfural-D	620-02-0	1601.8	1255.005	1.48434	spices, caramel wood
4	(E, E)-2,4-Heptadienal	4313-03-5	1515.7	1011.548	1.1746	fatty, oily, aldehyde
5	(E)-2-Octenal	2548-87-0	1452.3	863.1	1.3336	fresh cucumber, fatty, green herbal
6	Nonanal	124-19-6	1414.4	784.875	1.50685	rose, citrus, strong oily
7	(E)-2-Hexenal	6728-26-3	1214.9	452.581	1.53351	green, banana, fat
8	(E)-2-Pentenal	1576-87-0	1150.6	364.707	1.12189	potato, peas
9	Hexanal	66-25-1	1091.3	295	1.55822	fresh, green, fat
10	Pentanal	110-62-3	990.9	217.305	1.42322	green grassy, faint

11	3-Methylbutanal-M	590-86-3	932.4	182.071	1.15266	banana, pungent chocolate, fat
12	3-Methylbutanal-D	590-86-3	932.4	182.071	1.41714	chocolate, fat
13	Propanal	123-38-6	799.6	133.654	1.14152	pungent, green grassy
14	2-Methylpropanal	78-84-2	811.5	137.25	1.28136	banana, melon, slightly nutty
15	Acetaldehyde	75-07-0	735.0	115.678	0.98749	green, slight fruity
16	(E)-2-Nonenal	18829-56-6	1542.8	1082.486	1.40498	fatty, green, waxy
17	Heptanal	111-71-7	1186.6	413.966	1.33322	fresh, aldehyde, fatty
18	Butanal	123-72-8	885.9	162.092	1.28079	pungent, fruity, green leaf
Ketones						
1	2-Cyclohexen-1-one	930-68-7	1434.9	826.34	1.11422	roasted
2	alpha-Thujone	546-80-5	1408.1	772.565	1.36555	thujonic, menthol
3	2-Nonanone	821-55-6	1408.8	773.861	1.4362	fresh, sweet, green
4	6-Methyl-5-hepten-2-one	110-93-0	1351.7	670.847	1.1756	citrus, fruity, mouldy
5	5-Nonanone	502-56-7	1330.1	635.5	1.34308	special aromatic aroma
6	1-Hydroxy-2-propanone	116-09-6	1315.0	611.908	1.23354	pungent, caramel, fresh
7	1-Octen-3-one	4312-99-6	1314.2	610.757	1.28769	strong earthy, mushroom, vegetable
8	3-Hydroxy 2-butanone	513-86-0	1293.5	579.684	1.34185	butter, cream
9	2-Octanone	111-13-7	1303.2	594.07	1.78496	mouldy, ketone, milk
10	3-Octanone	106-68-3	1267.6	534.245	1.29376	mouldy, ketone, green
11	2-Heptanone	110-43-0	1181.5	407.346	1.62378	pear, banana, fruity
12	2,3-Pentanedione	600-14-6	1052.3	261.63	1.19712	sweet, cream, caramel
13	4-Methyl-2-pentanone	108-10-1	1015.8	234.323	1.18306	ketone
14	1-Penten-3-one	1629-58-9	1000.6	223.777	1.3148	strong pungent odors
15	2-Pentanone	107-87-9	988.0	215.387	1.36648	acetone, fresh,

16	2,3-Butanedione	431-03-8	958.7	197.171	1.1861	sweet fruity butter, popcorn, sweet taste
17	2-Butanone	78-93-3	916.8	173.682	1.24386	fruity, camphor
18	Acetone	67-64-1	831.3	143.482	1.11517	fresh, apple, pear
Acids						
1	Butanoic acid	107-92-6	1788.7	2004.186	1.16964	strong acetic acid, cheese, butter
2	2-Methylpropanoic acid	79-31-2	1667.4	1479.123	1.18212	yogurt, rancid cream
3	Propanoic acid	79-09-4	1530.3	1049.262	1.10927	yogurt, vinegar
Esters						
1	Diethyl succinate	123-25-1	1750.8	1822.588	1.29291	fruity
2	Ethyl decanoate	110-38-3	1707.9	1636.923	1.62507	fruity, wine, pear
3	Bornyl acetate	76-49-3	1652.1	1423.356	1.21816	herbal, pine leaf
4	gamma-Butyrolactone-M	96-48-0	1570.5	1160.307	1.08369	cream, fat, caramel
5	gamma-Butyrolactone-D	96-48-0	1570.9	1161.507	1.30526	cream, fat, caramel
6	Isobutyl acetate	110-19-0	1010.0	230.248	1.20941	fruity, raw pear, raspberrie
7	Propyl acetate	109-60-4	980.1	210.354	1.47794	fruity, pear
8	Ethyl acetate	141-78-6	894.0	165.053	1.33506	fresh, fruity, sweet
9	Ethyl propanoate	105-37-3	971.0	204.598	1.15471	grape, pineapple, fruity
Hydrocarbons						
1	(E)-beta-Farnesene	18794-84-8	1735.2	1752.631	1.46092	wood, citrus, sweet
2	Aromadendrene	489-39-4	1685.3	1546.736	1.46224	wood, fruit
3	beta-Elemene	515-13-9	1632.7	1356.016	1.46493	sweet, herb
4	gamma-Terpinene	99-85-4	1244.5	496.833	1.21504	oil, wood, terpenes
5	alpha-Terpinene-M	99-86-5	1174.0	396.594	1.21431	woody, lemon, citrus
6	alpha-Terpinene-D	99-86-5	1174.6	397.335	1.72481	woody, lemon, citrus
7	Myrcene	123-35-3	1161.7	379.538	1.66032	must, spice, balsamic
8	beta-Pinene	127-91-3	1116.8	323.18	1.21324	resin, green
9	Camphene	79-92-5	1069.6	275.72	1.21217	woody, camphor
10	alpha-Pinene-M	80-56-8	1024.5	240.555	1.21549	fresh, camphor, sweet

11	alpha-Pinene-D	80-56-8	1024.2	240.315	1.6644	fresh, camphor, sweet
12	Limonene	138-86-3	1193.7	423.325	1.21787	lemon, sweet, orange
Aromatic compounds						
1	Acetophenone-M	98-86-2	1805.3	2089.115	1.20027	sweet, spicy, almond
2	Acetophenone-D	98-86-2	1805.2	2088.835	1.57945	sweet, spicy, almond
3	Phenylacetaldehyde	122-78-1	1708.5	1639.575	1.25289	hyacinth, sweet fruity, almond
4	Benzaldehyde-M	100-52-7	1489.2	946.622	1.15066	bitter almond, cherry, nutty
5	Benzaldehyde-D	100-52-7	1489.2	946.622	1.4705	bitter almond, cherry, nutty
6	p-Cymene	99-87-6	1285.7	565.631	1.19462	fresh, citrus, terpene
Other compounds						
1	2-Acetylpyrazine	22047-25-2	1713.7	1660.796	1.14386	roast, popcorn, nutty
2	1H-Pyrrole	109-97-7	1497.3	966.111	0.97378	sweet, fruity, nuts
3	2-Acetyl furan	1192-62-7	1483.3	932.702	1.44385	fatty, sweet, caramel
4	Diallyl disulfide-M	2179-57-9	1459.1	877.948	1.19912	stinky, garlic
5	Diallyl disulfide-D	2179-57-9	1458.7	877.02	1.63648	stinky, garlic
6	2,5-Dimethylpyrazine	123-32-0	1332.6	639.528	1.49201	nutty, peanut, mouldy
7	2-Methylpyrazine	109-08-0	1272.6	542.857	1.38616	nutty, mouldy, roast
8	2-Pentylfuran	3777-69-3	1233.5	479.867	1.25155	bean, fruity, earthy
9	Diallyl sulfide	592-88-1	1134.2	343.943	1.33253	garlic
10	2,5-Dimethylfuran	625-86-5	945.2	189.262	1.37256	meaty, roast beef, bacon
11	Dimethyl sulfide	75-18-3	770.6	125.265	0.95608	cabbage, sulfur, gasoline
12	Tetrahydrofuran	109-99-9	877.1	158.935	1.22743	ether

Note: Suffixes "M" and "D" denote monomer and dimer, respectively. Elevated analyte concentrations may induce proton/electron sharing among multiple molecules within the IMS ionization region, leading to the formation of polymeric ions (e.g., dimers or trimers). These ions, despite originating from identical compounds (as evidenced by congruent retention time), exhibit distinct drift time during migration. Odor descriptors were curated from the following databases: http://www.odour.org.uk, http://www.flavornet.org, https://www.femaflavor.org/flavor-library,

and http://thegoodscentscompany.com.
