# Supplementary figures and images for: Evolution of Physicochemical Properties and Volatile Organic Compound Profiles in Pre-Cooked Braised Chicken During Storage
Source: Foods. 2025 Dec 29;15(1):91. doi: 10.3390/foods15010091 (PMC12785967; doi:10.3390/foods15010091)

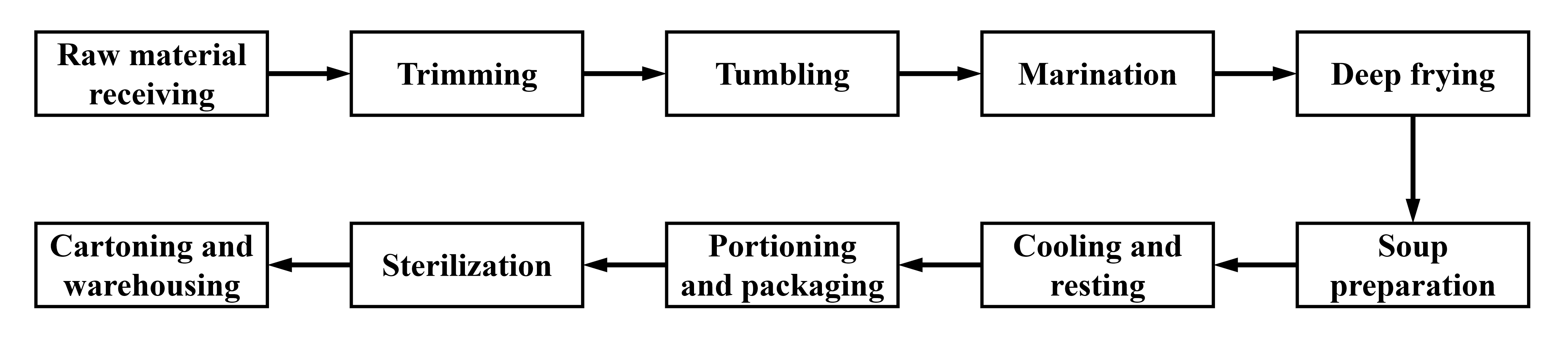

Supplement: Supplementary file 1 [file foods-15-00091-s001.zip › Figure S1.png]

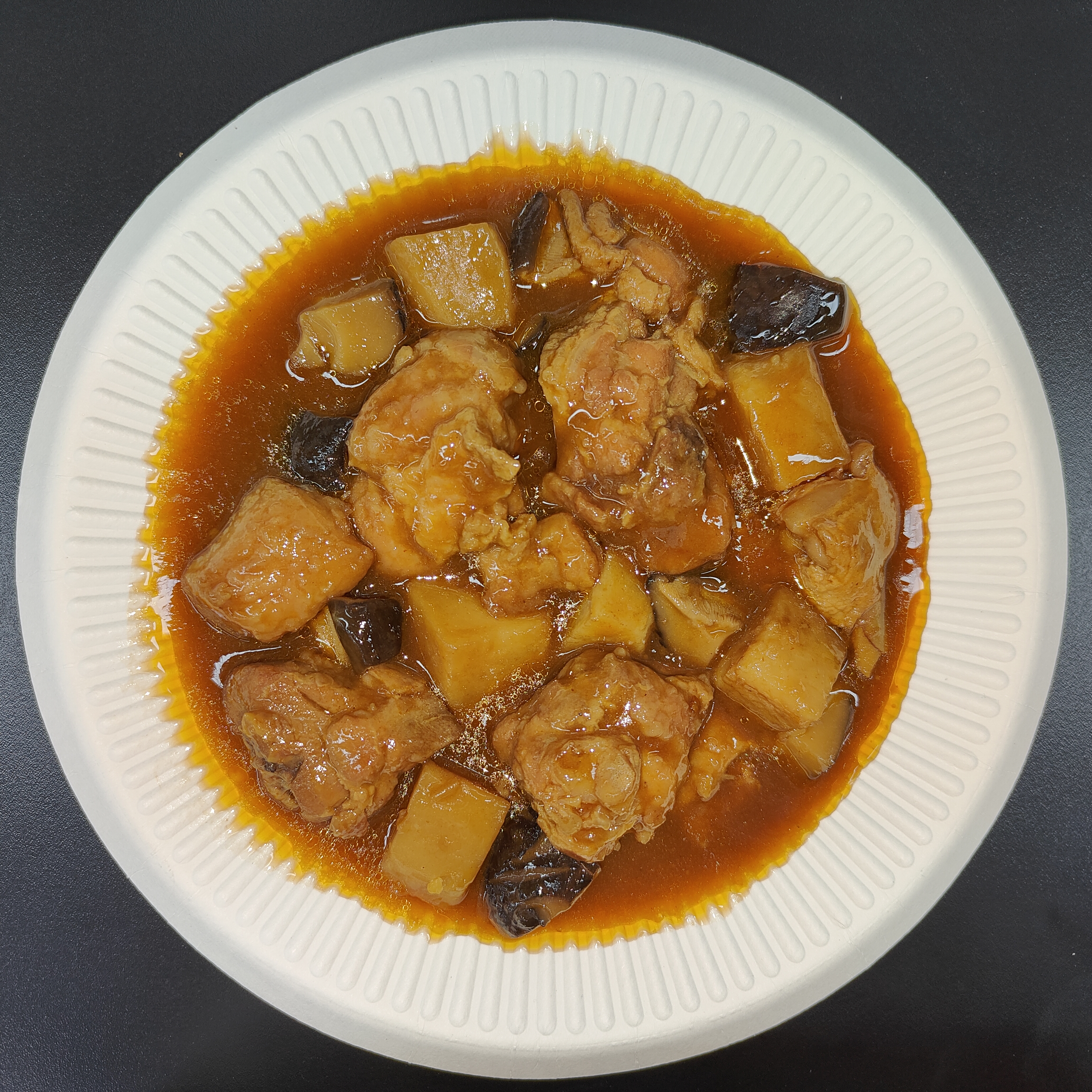

Supplement: Supplementary file 1 [file foods-15-00091-s001.zip › Figure S2.jpg]
